# Multi-omics reveals deoxycholic acid modulates bile acid metabolism via the gut microbiota to antagonize carbon tetrachloride-induced chronic liver injury

**DOI:** 10.1080/19490976.2024.2323236

**Published:** 2024-02-28

**Authors:** Li Zhang, Zhiyi Zheng, Huanhuan Huang, Ya Fu, Tianbin Chen, Can Liu, Qiang Yi, Caorui Lin, Yongjun Zeng, Qishui Ou, Yongbin Zeng

**Affiliations:** a Department of Laboratory Medicine, Gene Diagnosis Research Center, The First Affiliated Hospital, Fujian Medical University, Fuzhou, China; b Department of Laboratory Medicine, National Regional Medical Center, Binhai Campus of the First Affiliated Hospital, Fujian Medical University, Fuzhou, China; c Fujian Key Laboratory of Laboratory Medicine, The First Affiliated Hospital, Fujian Medical University, Fuzhou, China; d Fujian Clinical Research Center for Laboratory Medicine of Immunology, The First Affiliated Hospital, Fujian Medical University, Fuzhou, China; e Department of Pediatrics, The First Affiliated Hospital, Fujian Medical University, Fuzhou, China; f Department of Cardiology, The First Affiliated Hospital, Fujian Medical University, Fuzhou, China

**Keywords:** Chronic liver injury, gut microbiota, metabolism, transcriptome, inflammation, deoxycholic acid

## Abstract

Deoxycholic acid (DCA) serves essential functions in both physiological and pathological liver processes; nevertheless, the relationship among DCA, gut microbiota, and metabolism in chronic liver injury remain insufficiently understood. The primary objective of this study is to elucidate the potential of DCA in ameliorating chronic liver injury and evaluate its regulatory effect on gut microbiota and metabolism via a comprehensive multi-omics approach. Our study found that DCA supplementation caused significant changes in the composition of gut microbiota, which were essential for its antagonistic effect against CCl_4_-induced chronic liver injury. When gut microbiota was depleted with antibiotics, the observed protective efficacy of DCA against chronic liver injury became noticeably attenuated. Mechanistically, we discovered that DCA regulates the metabolism of bile acids (BAs), including 3-epi DCA, Apo-CA, and its isomers 12-KLCA and 7-KLCA, IHDCA, and DCA, by promoting the growth of *A.muciniphila* in gut microbiota. This might lead to the inhibition of the IL-17 and TNF inflammatory signaling pathway, thereby effectively countering CCl_4_-induced chronic liver injury. This study illustrates that the enrichment of *A. muciniphila* in the gut microbiota, mediated by DCA, enhances the production of secondary bile acids, thereby mitigating chronic liver injury induced by CCl_4_. The underlying mechanism may involve the inhibition of hepatic IL-17 and TNF signaling pathways. These findings propose a promising approach to alleviate chronic liver injury by modulating both the gut microbiota and bile acids metabolism.

## 1. Introduction

Chronic liver disease (CLD) is characterized by persistent hepatic injury and the ensuing pathological changes that occur within the liver over an extended period.^1^ Initially, the liver can partially regenerate in response to hepatitis-induced hepatocellular injury. However, persistent liver inflammation triggers a cascade of events leading to liver fibrosis. If left untreated, this progression can result in advanced liver cirrhosis and an increased susceptibility to hepatocellular carcinoma.^2^ Therefore, the crucial importance of early identification and intervention in the initial phases of CLD cannot be overstated, as they significantly contribute to reducing disease progression and limiting the occurrence and advancement of hepatocellular carcinoma.

Recent investigations have unveiled the intricate interplay between CLD and the metabolism of bile acids (BAs).^3^ BAs, derived from cholesterol through hepatic synthesis, fulfill pivotal roles in governing cholesterol metabolism and facilitating lipid absorption.^4^ Among the myriad of bile acids, deoxycholic acid (DCA) serves as the predominant constituent of secondary BAs. Following its gut synthesis, DCA undergoes reabsorption into the portal vein and subsequent hepatic recirculation via systemic circulation. This active uptake by hepatic cells contributes to the enterohepatic circulation of BAs.^5^ DCA is known for its strong antioxidant properties and its significant role in emulsification and solubilization processes. However, its exact contribution to the development and advancement of liver diseases remains unclear.

Recent research has focused on the beneficial effects of DCA in the context of liver diseases. For instance, the supplementation with DCA has been shown to ameliorate liver injury and mitigate inflammation in a murine model of *Klebsiella pneumoniae*-induced liver abscess and bacteremia.^6^ Additionally, a novel nanocomposite comprising DCA-grafted chitosan and oleanolic acid has demonstrated a hepatoprotective effect against carbon tetrachloride (CCl_4_)-induced liver injury.^7^ Clinical studies have also revealed diminished DCA levels in patients suffering from CLD of various etiologies, in comparison to healthy individuals.^8^ Nevertheless, the precise mechanism underlying the beneficial effects of DCA remains incompletely elucidated, necessitating further investigations to unravel the mechanisms by which DCA influences the development, progression, and therapeutic interventions of liver diseases.

The expanding field of omics technology and extensive scholarly research have illuminated the pivotal role of gut microbiota in the etiology and progression of liver disease. The close anatomical and physiological connection between the gut and the liver establishes the “gut-liver axis,” which enables communication through the bile duct, portal vein, and systemic circulation.^9^ On one hand, disruptions in the gut barrier permit the translocation of inflammatory mediators and harmful microbial metabolites into the liver through the portal vein, triggering the release of pro-inflammatory factors and ultimately leading to liver fibrosis.^10^ On the other hand, specific pathophysiological changes after liver injury, such as immune system impairment, inflammation, or disruptions in bile acid metabolism, may result in gut mucosal damage and modifications in the gut microbiota’s composition.^11^

Recent research findings have indicated that liver injury leads to a reduction in BAs levels, which in turn promotes the proliferation of gut microbiota. Importantly, this effect can be mitigated through interventions involving bile acids.^12^ However, it is essential to note that the exploration of the antagonistic influence of DCA on liver injury, particularly through microbiological and metabolomic approaches, remains somewhat limited in scope. Therefore, the primary objective of this study is to investigate the potential of DCA in alleviating CCl_4_-induced chronic liver injury by modulating bile acid metabolism through the gut microbiota, with the aim of providing a theoretical basis for developing new therapies for CLD.

## 2. Materials and methods

### 2.1. Study participants

#### 2.1.1. CLD patients and healthy control individuals

This study enrolled participants from July 2021 to November 2022 at the First Affiliated Hospital, Fujian Medical University. The recruited participants consisted of 10 individuals diagnosed with nonalcoholic steatohepatitis (NASH), 10 individuals with alcoholic liver disease (ALD), 10 individuals with primary biliary cholangitis (PBC), and 20 healthy control (HC) individuals, all matched for age and gender. The diagnosis of CLD was established based on the criteria outlined in the guidelines published by the American Association for the Study of Liver Diseases (AASLD).^13–16^ The blood samples were collected and processed in strict accordance with standard operating procedures for serum collection.^17^

Routine blood testing conducted using the Siemens ADVIA 2120i Hematology Analyzer. Routine biochemistry was assayed using automated biochemical technique (Siemens Healthcare Diagnostics), while coagulation function was measured using a fully automatic blood coagulation analyzer (CS5100, Sysmex Corp, Japan).

#### 2.1.2. Experimental animals

Male C57BL/6 mice of specific pathogen-free (SPF) grade, weighing between 20–22 g, were obtained from Weitong Lihua Experimental Animal Technology Co., Ltd (Production License No: SCXK (Zhejiang) 2019–0001). Chronic liver injury in mice was induced by repeated administration of CCl_4_ according to the procedure reported in the literature.^18^ Antibiotic cocktail consisted of ampicillin (2.5 g/L), metronidazole (1.25 g/L), neomycin (2.5 g/L), gentamicin (1.25 g/L) and vancomycin (1.25 g/L).

**Figure 1.**
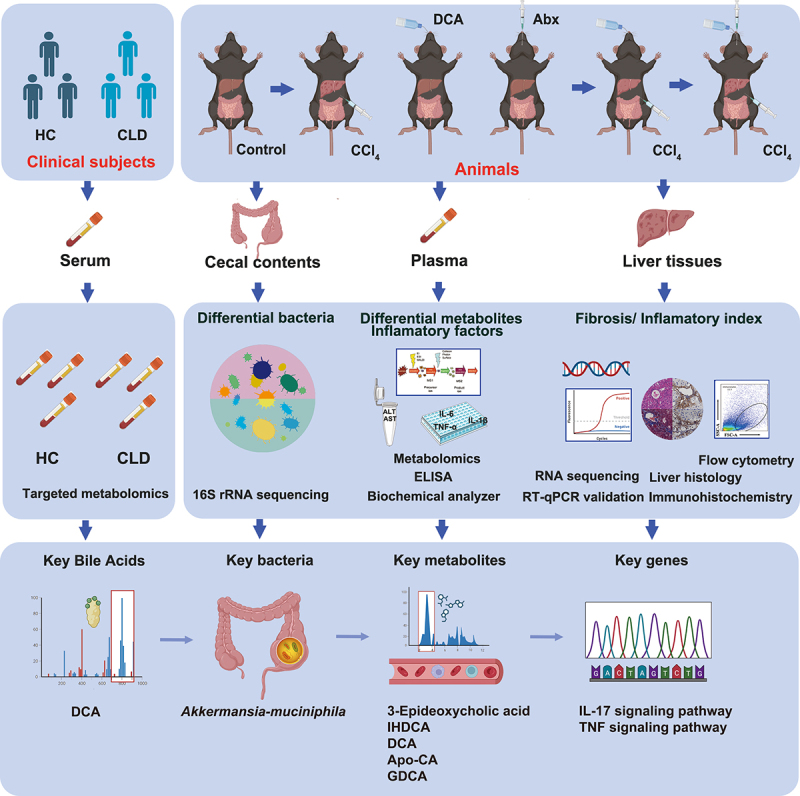
Experimental design and procedure HC=Healthy control; CLD=Chronic liver disease; DCA=Deoxycholic acid; Abx=Antibiotic cocktail.

A total of 36 male C57BL/6J mice were randomly divided into six groups: the control group (Control), model group (CCl_4_), DCA and CCl_4_ treatment group (CCl_4_+DCA), DCA, CCl_4_ and antibiotic cocktail treatment group (CCl_4_+DCA+Abx), antibiotic cocktail treatment group (Abx), and DCA treatment group (DCA). Each group comprised of six mice. The control group mice were fed a normal diet. The CCl_4_ group mice received intraperitoneal injections of CCl_4_ (diluted 1: 40 in olive oil, 2.5%, 5 μL/g) for five weeks. The CCl_4_+DCA group received drinking water containing 0.2% DCA (0.2 grams of sodium deoxycholate powder dissolved in 100 mL of sterile drinking water) for three days prior to CCl_4_ injection and continued until the end of the five-week period after CCl_4_ injection. The CCl_4_+DCA+Abx group mice were given an antibiotic cocktail by gavage for 9 days before DCA treatment and continued until the end of the five-week period of CCl_4_ injection. At the conclusion of the experimental period, the mice were euthanized, and samples of their plasma, liver, and cecal contents were collected for subsequent analysis. Figure 1 depicts the study design process, including detailed grouping information and experimental workflow.

### 2.2. Ethics

The study was approved by the Ethics Committee of the First Affiliated Hospital of Fujian Medical University (MTCA, ECFAH of FMU [2021] 047). Written informed consent was obtained from each patient. Animal experiments were approved by the Animal Ethics Committee of Fujian Medical University (IACUC FJMU 2021–0307).

### 2.3. Detection of bile acids

In the present study, a total of 65 different bile acids were analyzed. Bile acids contents were detected by MetWare (http://www.metware.cn/) based on the AB SCIEX QTRAP 6500 LC-MS/MS platform. Sample preparation and extraction were as follows: samples (50 μL) were extracted with 200 μL methanol/acetonitrile (V/V = 2:8). A 10 μL internal standard mixed solution (1 μg/mL) (Table S1) was introduced into the extract as internal standards for quantification. The samples were then placed at −20°C for 10 minutes to precipitate proteins. Subsequently, after centrifugation for 10 minutes at 12,000 rpm and 4°C, the supernatant was transferred to clean plastic microtubes. The extracts were evaporated to dryness and reconstituted in 100 μL 50% methanol (V/V).

Qualitative and quantitative MS analysis of the metabolites in the samples was conducted using the self-built MetWare database (MWDB) (MetWare Company, Wuhan, China) and multiple reaction monitoring (MRM). The mass spectrometry platform identified BAs with molecular weights ranging from 73.8 to 594.248 Daltons.

The HPLC conditions and ESI-MS/MS conditions are shown in the Supplementary document.

### 2.4. Plasma metabolite analysis

Plasma metabolites were analyzed using the UPLC-MS/MS platform, employing a widely targeted metabolomics analysis. This approach initially identifies metabolites in the sample through non-targeted metabolomics technology, followed by precise quantitative detection of metabolites based on widely targeted metabolomics analysis (performed by Wuhan MetWare Biotechnology Co., Ltd).

Sample preparation and extraction were as follows: The sample stored at −80°C in the freezer was thawed on ice and vortexed for 10 seconds. Subsequently, 50 μL of the sample and 300 μL of an extraction solution (ACN: Methanol = 1:4, V/V) containing internal standards (Table S2) were added to a 2 mL microcentrifuge tube. The sample was vortexed for 3 minutes and then centrifuged at 12,000 rpm for 10 minutes (4°C). A total of 200 μL of the supernatant was collected, placed at −20°C for 30 minutes to precipitate proteins., and then centrifuged at 12,000 rpm for 3 minutes (4°C). Aliquots of 180 μL of the supernatant were transferred for LC-MS analysis.

The data acquisition instrument system for non-targeted detection mainly consists of UPLC and Quadrupole Time of Flight (Triple TOF 6600, AB SCIEX). The data collection instrument system for widely targeted detection mainly utilizes an LC-ESI-MS/MS system (UPLC, ExionLC AD, https://sciex.com.cn/; MS, QTRAP®6500+System, https://sciex.com/). The pooled sample was prepared by combining equal aliquots of the supernatants from all samples，and was served as a reference for quality control (QC) purposes throughout the analysis. Accurate qualitative analysis of metabolites was conducted based on the self-built MWDB database (including secondary mass spectrometry and retention time) established by Wuhan MetWare Biotechnology Co., Ltd, as well as public databases (including METLIN, KEGG, and HMDB), CFM-ID 3.0 web server (https://cfmid.wishartlab.com/), and MetDNA.^19,20^

The HPLC conditions, QTOF-MS/MS, and ESI-Q TRAP-MS/MS conditions are shown in the Supplementary document.

### 2.5. 16S rRNA microbial community analysis

The 16S rRNA gene variable region, specifically the V3-V4 region, present in the cecal contents of mice, was amplified and subjected to next-generation sequencing (NGS) for comprehensive analysis. The DNA extraction was performed using the DNeasy PowerSoil Kit (Qiagen), and the quality of the extracted DNA was evaluated using 1% agarose gels. The amplification of the targeted 16S rRNA gene region was carried out using Pyrobest DNA polymerase (TaKaRa). The resulting PCR products were used for the construction of the sequencing library, following the established protocols outlined in the Illumina TruSeq DNA Sample Preparation Guide. The library was quantified using Pico green and a fluorescence spectrophotometer. Quality control of the PCR-enriched fragments was performed using the Agilent 2100 system to validate the size and distribution of the DNA library fragments. Finally, the library was subjected to sequencing on an Illumina platform. Sequencing service was provided by the Personal Biotechnology Co., Ltd. Shanghai, China.

### 2.6. Flow cytometry analysis of liver inflammatory cells

To prepare a single cell suspension from 150 mg of liver tissue, we utilized the Fresh/Frozen Tissue Single Cell Suspension Separation Kit (Minute™) according to the manufacturer’s instructions. Incubate the non-parenchymal cell precipitates with 1 mL of red blood cell lysate at room temperature for 5 minutes. Add 10 mL of phosphate-buffered saline (PBS) containing 5% fetal bovine serum (FBS) and mix well. Centrifuge the cell suspension at 900 rpm for 7 minutes at 4°C. Count the centrifuged cells and divide them into tubes with 10^6^ cells per tube, resuspending them in 100 μL of PBS containing 5% FBS. The released cells were stained with FITC-conjugated anti-mouse CD45 (Thermo Fisher Scientific, Clone: 30-F11), PE-conjugated anti-mouse CD11b (Thermo Fisher Scientific, Clone: M1/70), APC-conjugated anti-mouse F4/80 (Thermo Fisher Scientific, Clone: BM8), PE/Cy5-conjugated anti-mouse Ly-6 G (Thermo Fisher Scientific, Clone: 1A8-Ly6g) and PE/Cy7-conjugated anti-mouse Ly-6C (Thermo Fisher Scientific, Clone: HK1.4) with a 1:100 diluted solution. Wash the cells with PBS containing 5% FBS by centrifuging them at 650 g for 5 minutes at 4°C, remove the supernatant, and resuspend the cells in 300 μL of sheath fluid. Pass the cell suspension through a 70-μm cell sieve and transfer it into a flow tube. Use flow cytometry channels for CD45-FITC, CD11b-PE, Ly6C-PE/Cy7, Ly6G-PE/Cy5, and F4/80-APC to observe the infiltration of inflammatory cells. The gating strategy used for the flow cytometry analysis is shown in the Supplementary Figure S1.

### 2.7. Quantification of plasma inflammatory biomarkers

Peripheral blood samples were collected from mice, and 1.5 g/dL of Na_2_EDTA was used as an anticoagulant. Plasma fractions were prepared by centrifuging the samples at 2–8°C and 1000 g for 15 minutes within 30 minutes of collection. The instructions provided with each ELISA kit (Elabscience) for mouse IL-6, IL-1β, and TNF-α were strictly followed.

### 2.8. Histological staining

The liver tissues were fixed in 10% neutral buffered formalin for 48 h. After dehydration, the tissues were embedded in paraffin and sliced (5 mm) by Rotary Microtome (Servicebio Technology Co., Ltd, Wuhan). The tissue slices underwent dewaxing using xylene and rehydration with ethanol. Hematoxylin-Eosin (H&E) (Servicebio Technology Co., Ltd, Wuhan) and Masson (Servicebio Technology Co., Ltd, Wuhan) staining was performed for histological examination. For immunohistochemical staining, antigen retrieval was conducted using citric acid. Endogenous peroxidase activity was blocked with hydrogen peroxide. The slices were then incubated with primary and secondary antibodies (Cell Signaling Technology, USA), and antibody binding was visualized using DAB chromogenic solution (Servicebio Technology Co., Ltd, Wuhan). Hematoxylin counterstaining was applied to nuclear visualization.

### 2.9. RNA extraction and real-time qPCR

Total RNA of liver tissues was isolated using SteadyPure Rapid RNA extraction Kit (Accurate Biology, China) according to the manufacturer’s instruction. RNA was reverse transcribed into cDNA using the HiScript III RT SuperMix for qPCR (+gDNA wiper) Reverse Transcription kit (Vazyme, Jiangsu, Nanjing, China) according to the manufacturer’s instructions. 20 μL PCR reactions were prepared using the Taq Pro Universal SYBR qPCR Master Mix qPCR reaction specific premix (Vazyme, Jiangsu, Nanjing, China), and qPCR was carried out using ABI 7500 Real-Time PCR System. Specific mouse primer sequences (Sangon Biotech, China) are provided in Table S3. β-actin expression was used as an internal control.

### 2.10. Data and statistical analysis

Results were presented as frequency and percentage for categorical variables, mean ± standard deviation (SD) for normally distributed continuous variables and median (interquartile range) for not normally distributed continuous variables. Data analysis and statistical plotting were performed using *R* language (version 4.1.1), *SPSS* software (version 26.0), and *GraphPad Prism* software (version 9.0). The normality of each data group was assessed using the *One Sample Kolmogorov-Smirnov* test. For comparisons between two independent samples, either the *t*-test or *Wilcoxon* rank sum test was employed. Differences among multiple groups were assessed using *one-way ANOVA* or the *Kruskal-Wallis* test. Categorical variables were compared using the chi-square test. Two-way orthogonal partial least squares (O2PLS) analysis was performed to integrate transcriptomic and metabolomic data. *Spearman’s* correlation analysis was applied to assess the correlation between variables. *P*-value <0.05 was considered statistically significant.

#### 2.10.1. Analysis of 16S rRNA sequencing

16S rRNA gene sequencing data undergoes sequence denoising or OTU clustering and is then subjected to taxonomy composition analysis using the analysis methods provided by the QIIME2 software (version 2019.4) and the Vsearch software. The “qiime diversity alpha rarefaction” command was employed to calculate alpha diversity indices (Chao1, observed species, Shannon) to compare bacterial richness and diversity across different samples. Beta diversity analysis was performed by calling the “qiime diversity core-metrics-phylogenetic” or “qiime diversity core-metrics” command through QIIME2 (version 2019.4) utilizing the ape package in R. Bray-Curtis distance matrix was calculated, and Principal Coordinate Analysis (PCoA) was conducted based on this matrix. The LEfSe (LDA Effect Size) analysis, employing the Python LEfSe package, R language, and ggtree package, integrates nonparametric Kruskal-Wallis and Wilcoxon rank sum tests with Linear discriminant analysis (LDA) effect size estimation, setting the LDA threshold to 2.

#### 2.10.2. Analysis of transcriptome sequencing

The reference genomes and gene model annotation files were obtained from the genome website. For expression analysis, HTSeq (version 0.9.1) was used to statistically compare the Read Count values of each gene, which served as the baseline expression level. The expression levels were further standardized using FPKM. Differential expression analysis between two comparative combinations was performed using DESeq software (version 1.20.0). Volcano plots of differentially expressed genes were generated using the ggplot2 software package in R. Cluster analysis was conducted using the Pheatmap software package in R to perform bidirectional clustering analysis on the union of differentially expressed genes and samples across all comparative groups. KEGG pathway enrichment analysis was carried out using the Cluster Profiler software (version 3.4.4), with a focus on identifying significantly enriched pathways with a *P*-value <0.05. Gene set enrichment analysis (GSEA) and visualization were performed using the FGSEA package in R.

#### 2.10.3. Metabolomics analysis

Unsupervised Principal component analysis (PCA) was performed by statistics function prcomp within *R* (www.r-project.org). The Hierarchical cluster analysis (HCA) results of samples and metabolites were presented as heatmaps with dendrograms, while Pearson correlation coefficients between samples were calculated by the cor function in *R* and presented as only heatmaps. Both HCA and PCC were carried out by *R* package pheatmap. Significantly regulated metabolites between groups were determined by VIP (VIP ≥1) and absolute log_2_FC (|log_2_FC| ≥ 1). VIP values were extracted from the result of Orthogonal Partial Least Squares Discriminant Analysis (OPLS-DA), which also includes score plots and permutation plots, and was generated using the R package MetaboAnalystR. The data were log-transformed and mean-centered before OPLS-DA. To avoid overfitting, a permutation test (200 permutations) was performed. Identified metabolites were annotated using KEGG compound database (http://www.kegg.jp/kegg/compound/), annotated metabolites were then mapped to KEGG Pathway database (http://www.kegg.jp/kegg/pathway.html). Pathways with significantly regulated metabolites were then subjected to MSEA (metabolite sets enrichment analysis), and their significance was determined using hypergeometric test *P*-values.

## 3. Results

### 3.1. Bile acids profiles of CLD patients

**Figure 2.**
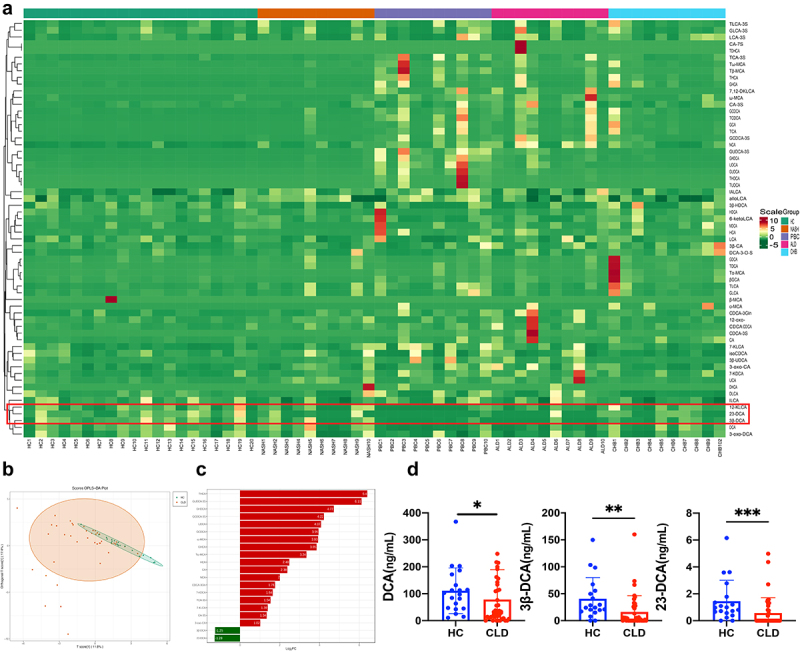
Characteristics of bile acids profiles in CLD patients (a) heatmap cluster analysis comparing CLD patients and healthy controls. The left cluster line represents the metabolite cluster, and the upper cluster line represents the sample cluster; (b) OPLS-DA score plot illustrating the differentiation between CLD patients and healthy controls; (c) bar chart depicting the differences in metabolite levels between CLD patients and healthy individuals; (d) histograms displaying the levels of 23-DCA, 3β-DCA, and DCA in CLD patients compared to healthy controls. HC: healthy control (*n* = 20); NASH: nonalcoholic steatohepatitis (*n* = 10); PBC: primary biliary cholangitis (*n* = 10); ALD: alcoholic liver disease (*n* = 10); CHB: chronic hepatitis B; CLD: chronic liver disease (*n* = 40), *P < .05, **P < .01, ***P < .001.

Table S4 presents the basic clinical information of study participants. As shown in Figure 2(a,b), the clustering thermogram and orthogonal partial least-squares discriminant analysis (OPLS-DA) demonstrated distinguishable differences in the BAs profiles between CLD patients with different etiologies and healthy controls (HC). As shown in Figure 2(c,d), the bar chart and histogram illustrating the differential metabolites highlight that CLD patients exhibit reduced levels of DCA, 23-nordeoxycholic acid (23-DCA), and 3β-deoxycholic acid (3β-DCA) compared to HC. Meanwhile, Supplementary Figure S2 provides a comprehensive analysis of the alterations in BAs levels observed in patients with various etiologies of CLD. This highlights the dysregulation of BAs metabolism in CLD patients, characterized by significant decreases in the levels of DCA, 23-DCA, and 3β-DCA. Table S5 presents the content of differential metabolites identified through comparative analysis between the HC and CLD groups.

### 3.2. DCA antagonizes the CCl_4_-induced liver inflammation via gut microbiota

#### 3.2.1. Histopathological examination reveals the protective role of DCA against CCl_4_-induced liver inflammation mediated by gut microbiota

**Figure 3.**
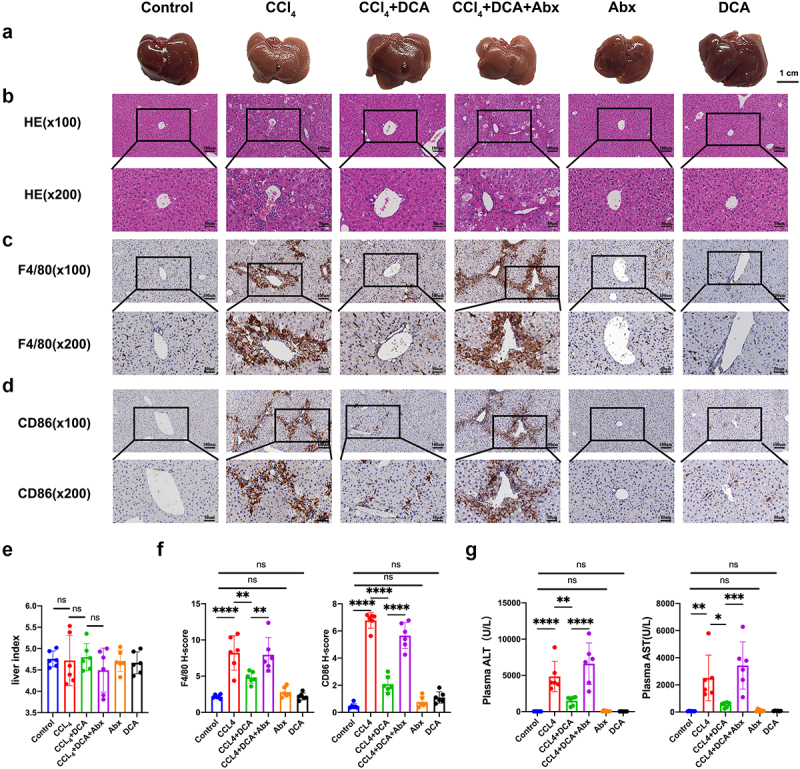
Effect of DCA on the pathological and plasma biochemical indexes of liver inflammation in mice (a) typical appearance of liver; (b) Representative liver histology by H&E staining (×100 and × 200); (c) immunohistochemical staining of F4/80 in liver tissue (×100 and × 200); (d) immunohistochemical staining of CD86 in liver tissue (×100 and × 200); (e) liver index of mice in different groups; (f) quantitative results of histochemical staining of F4/80 and CD86 in liver tissue. (g) Plasma ALT and AST levels. *n* = 6，*P < .05，**P < .01，***P < .001，****P < .0001. “ns” indicates no significant difference.

Liver surface morphology was examined in six groups of mice. The Control, DCA, and Abx groups exhibited a healthy, ruddy, and smooth liver surface, while the CCl_4_ model group and CCl_4_+DCA+Abx group showed a relatively pale liver color with rough and uneven surfaces. The mice in the CCl_4_+DCA group did not show liver appearance as favorable as those in the Control group but exhibited a redder appearance with fewer irregularities compared to the CCl_4_ model group and CCl_4_+DCA+Abx group. No significant changes in liver size were observed (Figure 3(a)).

Histological analysis using H&E staining showed no significant alterations in the Control, DCA, and Abx groups. However, the CCl_4_ model group exhibited morphological disruptions such as loss of liver cell morphology, enlarged nuclei, cytoplasmic loosening with central vacuolization, punctate necrosis, increased cytoplasmic eosinophilia, disappearance of cytoplasmic granules and nuclei, as well as inflammatory cell infiltration and aggregation in the portal area. In contrast, the CCl_4_+DCA group demonstrated a significant reduction in liver lesion severity, including decreased liver cell necrosis and reduced inflammatory cell presence in the portal area. Notably, depletion of gut microbiota in the CCl_4_+DCA+Abx group led to liver tissue alterations like those observed in the CCl_4_ model group, indicating a weakened inhibitory effect of DCA on liver inflammation following gut microbiota depletion (Figure 3(b)).

Immunohistochemical analysis revealed a significant increase in the expression of F4/80 and CD86 in liver tissue of the CCl_4_ model group, indicating an increase in macrophages, particularly type I macrophages (*p* < 0.0001) compared to the Control group. In contrast, the CCl_4_+DCA group showed a significant decrease in liver macrophages (*p* < 0.01) and type I macrophages (*p* < 0.0001) compared to the CCl_4_ group, suggesting the antagonistic effect of DCA against CCl_4_-induced macrophage increase. Interestingly, depletion of gut microbiota in the CCl_4_+DCA+Abx group led to a significant increase in liver macrophages (*p* < 0.01) and type I macrophages (*p* < 0.0001) compared to the CCl_4_+DCA group, indicating that the antagonistic effect of DCA on macrophage increase is significantly weakened after gut microbiota depletion (Figure 3(c,d,f)). No difference was seen among groups regarding the liver index (Figure 3(e)).

#### 3.2.2. DCA mitigates the expression of indicators of hepatocellular damage in plasma via modulation of the gut microbiota

Regarding indicators of hepatocellular damage in plasma, no significant changes in ALT and AST levels were observed in the Control, DCA, and Abx groups, indicating minimal impact on liver cell damage. In contrast, the CCl_4_ model group showed significantly elevated ALT and AST levels compared to the Control group (*p* < 0.0001, *p* < 0.01). The CCl_4_+DCA group exhibited a significant decrease in ALT and AST levels compared to the CCl_4_ group (*p* < 0.01, *p* < 0.05), demonstrating DCA’s ability to counteract the increase in plasma ALT and AST levels. However, the CCl_4_+DCA+Abx group displayed a notable increase in ALT and AST levels compared to the CCl_4_+DCA group (*p* < 0.0001, *p* < 0.001), indicating a substantial reduction in DCA’s effectiveness in mitigating the rise in plasma ALT and AST levels after gut microbiota depletion (Figure 3(g)).

#### 3.2.3. DCA reduces inflammatory cell population in the liver induced by gut microbiota, as analyzed by flow cytometry

**Figure 4.**
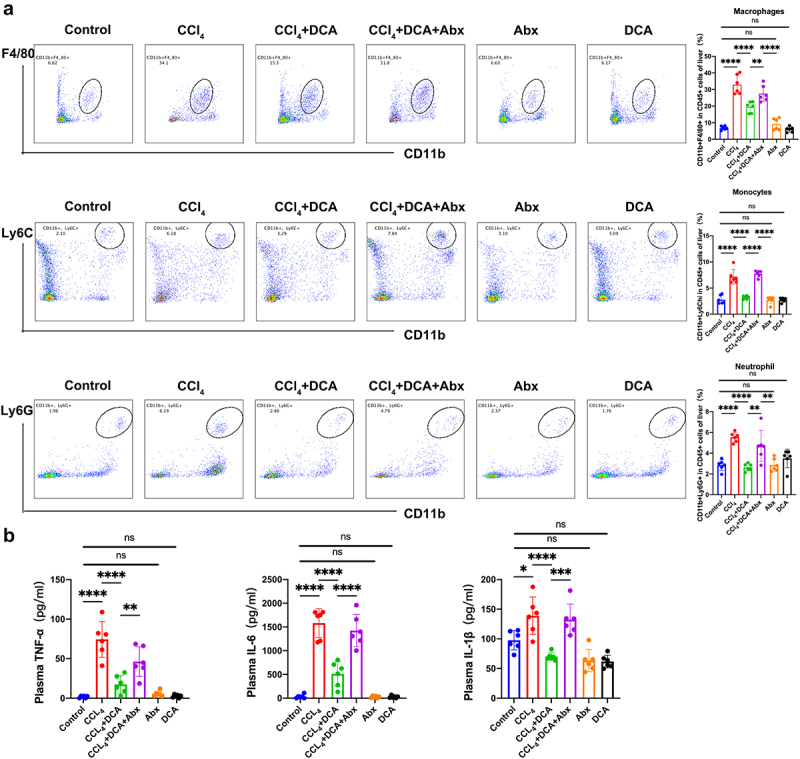
Effect of DCA on the hepatic inflammatory cells and plasma inflammatory factors in mice (a) detection of inflammatory cells in liver by flow cytometry [flow cytometry plot (left) and bar graph (right)]. Liver macrophages were identified as CD45+F4/80+CD11b+cells. Liver monocytes were identified as CD45+CD11b+Ly6C hi cells. Liver neutrophils were identified as CD45+Ly6G+CD11b+cells; (b) plasma levels of TNF-α, IL-6 and IL-1β. *n* = 6, *P < .05, **P < .01, ***P < .001, ****P < .0001. “ns” indicates no significant difference.

The Control, DCA, and Abx groups showed no significant changes in the inflammatory cell populations (including CD45+CD11b+F4/80+macrophages, CD45+CD11b+Ly6C hi monocytes, and CD45+CD11b+Ly6G+ neutrophils). The CCl_4_ group displayed a significant increase in inflammatory cells within the liver tissue compared to the Control group (*p* < 0.0001), indicating substantial infiltration due to CCl_4_ injection. Conversely, the CCl_4_+DCA group demonstrated a significant reduction in liver tissue inflammatory cells compared to the CCl_4_ model group (*p* < 0.0001), demonstrating the ability of DCA to counteract inflammatory cell infiltration in CCl_4_-induced chronic liver injury in mice. The CCl_4_+DCA+Abx group showed a notable increase in liver tissue inflammatory cells compared to the CCl_4_+DCA group (*p* < 0.01, *p* < 0.0001, *p* < 0.01), suggesting a significant attenuation of DCA’s inhibitory effect on liver inflammatory cell infiltration in mice following gut microbiota depletion (Figure 4(a)).

#### 3.2.4. DCA reduces the expression of plasma inflammatory factors by modulation of the gut microbiota

ELISA analysis of plasma inflammatory factors showed no significant changes in the control group, DCA group, and Abx group mice. The CCl_4_ model group exhibited a significant increase in the levels of IL-6, TNF-α, and IL-1β compared to the control group (*p* < 0.0001, *p* < 0.0001, *p* < 0.05). However, the CCl_4_+DCA group showed a significant decrease in the levels of these inflammatory factors compared to the CCl_4_ model group (*p* < 0.0001), indicating that DCA can counteract the increased expression of inflammatory factors induced by CCl_4_ in mice with chronic liver injury. The CCl_4_+DCA+Abx group exhibited a significant increase in the levels of IL-6, TNF-α, and IL-1β in liver tissue compared to the CCl_4_+DCA group (*p* < 0.0001, *p* < 0.01, *p* < 0.001), suggesting a substantial attenuation of DCA’s ability to counteract the increased expression of liver inflammatory factors in mice following gut microbiota depletion (Figure 4(b)).

### 3.3. DCA antagonizes CCl_4_-induced liver fibrosis via gut microbiota

**Figure 5.**
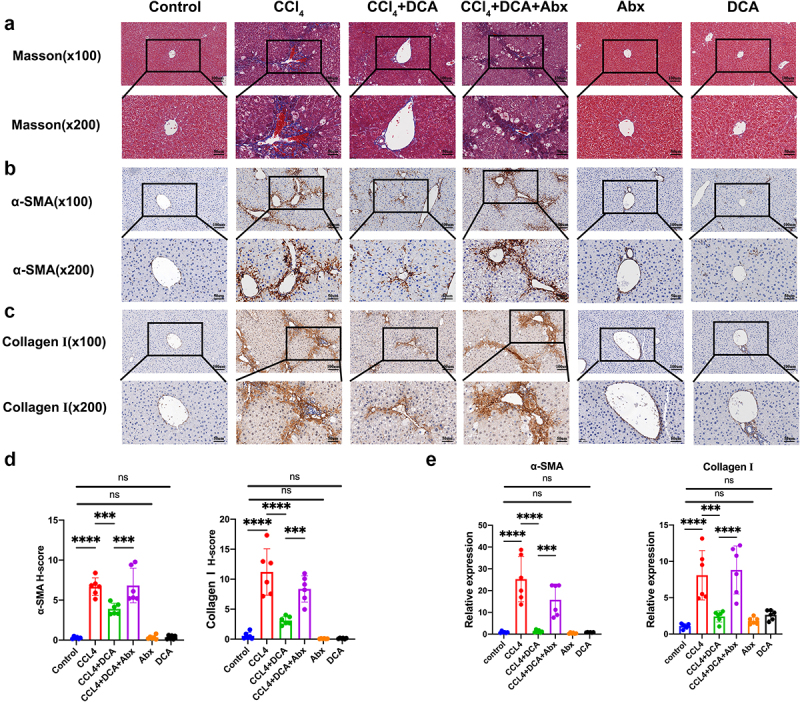
Effect of DCA on the pathological and mRNA expression of liver fibrosis in mice (a) Masson staining of liver tissue (×100 and × 200); (b) immunohistochemical staining of α-SMA (×100 and × 200); (c) immunohistochemical staining of collagen I in liver tissue (×100 and × 200); (d) quantitative results of histochemical staining for α-SMA and collagen I; (e) mRNA expression of α-SMA and collagen I in liver tissue. *n* = 6, *P < .05, **P < .01, ***P < .001, ****P < .0001. “ns” indicates no significant difference.

Masson staining revealed no significant fibrous hyperplasia in the Control, DCA, and Abx groups. However, the CCl_4_ group exhibited marked fibrous hyperplasia around the portal area. In contrast, the CCl_4_+DCA group showed a reduction in fiber proliferation and bridging, indicating a significant decrease in liver collagen fiber content due to DCA treatment. Interestingly, the CCl_4_+DCA+Abx group, characterized by gut microbiota depletion, displayed a significant increase in fiber proliferation around the portal area compared to the CCl_4_+DCA group (Figure 5(a)).

Immunohistochemical analysis of α-SMA and Collagen I protein expression showed no significant changes in the control, DCA, and Abx groups. The CCl_4_ group exhibited increased expression of α-SMA, and Collagen I compared to the control group (*p* < 0.0001, *p* < 0.0001). However, the CCl_4_+DCA group demonstrated decreased expression of α-SMA, and Collagen I compared to the CCl_4_ group (*p* < 0.001, *p* < 0.0001), indicating the ability of DCA to reduce their expression. Same as before, the CCl_4_+DCA+Abx group showed increased expression of α-SMA and Collagen I compared to the CCl_4_+DCA group (*p* < 0.001, *p* < 0.001), suggesting a weakened effect of DCA after gut microbiota depletion (Figure 5(b–d)).

In addition, we examined the mRNA expression levels of α-SMA and Collagen I in the liver. The CCl_4_ model group exhibited a significant increase in α-SMA, and Collagen I mRNA expression compared to the control group (*p* < 0.0001, *p* < 0.0001). However, the CCl_4_+DCA group showed a significant reduction in α-SMA, and Collagen I mRNA expression compared to the CCl_4_ group (*p* < 0.0001, *p* < 0.001), demonstrating the ability of DCA to significantly decrease their expression. Notably, in the CCl_4_+DCA+Abx group, there was a significant increase in α-SMA, and Collagen I mRNA expression compared to the CCl_4_+DCA group (*p* < 0.001, *p* < 0.0001), indicating a weakened effect of DCA on their mRNA expression following depletion of gut microbiota (Figure 5(e)).

### 3.4. DCA modulates the gut microbiota to antagonize CCl_4_-induced chronic liver injury

**Figure 6.**
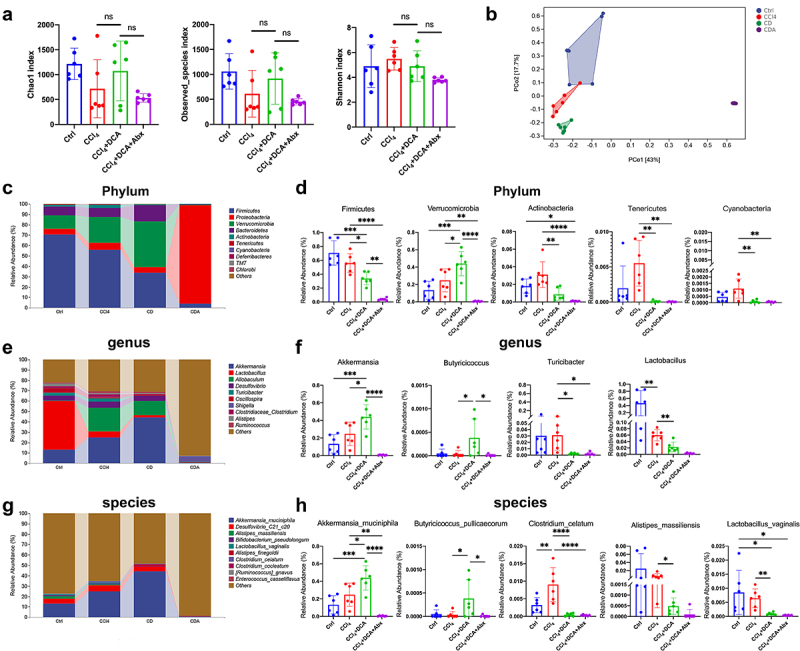
DCA modulates the composition of gut microbiota (a) α-diversity assessed using Chao1 Index, Observed_ species Index, and Shannon Index for each group; (b) Principal coordinate analysis (PCoA) of gut microbiota; (C) the relative abundance of the top 10 bacteria at the phylum level; (D) Representative histograms of gut microbiota at the phylum level; (E) the relative abundance of the top 10 bacteria at the genus level; (f) Representative histograms of gut microbiota at the genus level; (g) the relative abundance of the top 10 bacteria at the species level; (H) Representative histograms of gut microbiota at the species level. Ctrl: Control; CD: CCl_4_+DCA; CDA: CCl_4_+DCA+Abx. *n* = 6, *P < .05, **P < .01, ***P < .001, ****P < .0001. “ns” indicates no significant difference.

In the study, we performed 16S rRNA sequencing on cecal contents to elucidate the interplay between DCA, gut microbiota, and liver injury. Our analysis of α-diversity revealed no significant changes in microbial richness and diversity following DCA treatment (Figure 6(a)). However, PCoA analysis demonstrated notable alterations in microbial composition, as evidenced by distinct clustering patterns observed among the experimental groups (Figure 6(b)).

As shown in Figure 6(c–h) and Table S6, at the phylum level, the CCl_4_+DCA group exhibited a reduction in Firmicutes and an increase in Verrucomicrobiota, while Actinobacteria, Tenericutes, and Cyanobacteria decreased significantly compared to the CCl_4_ group (Figure 6(c,d)). At the genus level, the CCl_4_+DCA group showed increased abundance of Akkermansia and Butyricicoccus, which are negatively correlated with inflammation, while Turicibacter and Lactobacillus, associated with host inflammation, were significantly reduced (Figure 6(e,f)). Similarly, at the species level, the CCl_4_+DCA group demonstrated increased levels of *Akkermansia muciniphila* (*A.muciniphila*) and *Butyricicoccus pullicaecorum*, and decreased levels of *Alistipes massiliensis*, *Clostridium celatum*, and *Lactobacillus vaginalis* compared to the CCl_4_ group (Figure 6(g,h)).

**Figure 7.**
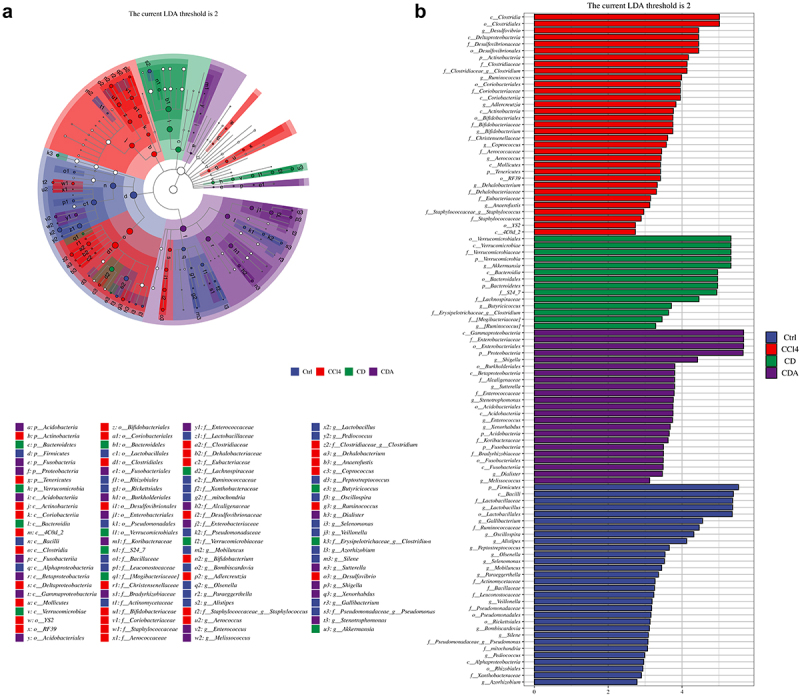
LEfse analysis of DCA modulating the composition of gut microbiota (a) the classification branch diagram illustrates the hierarchical relationship of main taxa from phylum to genus (from inner circle to outer circle) in the sample community. The node size corresponds to the average relative abundance of the taxon, and nodes of each color represent significant inter-group differences in these taxa. The abundance is higher in the group samples represented by the color; (b) the distribution bar chart of linear discriminant analysis (LDA) values for significantly different species demonstrates the significantly enriched species within each group and their importance. The LDA score reflects significant differences in bacteria among each group, and only bacteria meeting the significant LDA threshold of 2 are shown. The vertical axis represents taxonomic units with significant differences between groups, while the horizontal axis displays the logarithmic scores of LDA analysis for each taxonomic unit. Ctrl: Control; CD: CCl_4_+DCA; CDA: CCl_4_+DCA+Abx. *n* = 6.

Subsequently, we conducted additional analysis using Linear discriminant analysis effect size (LEfSe) to identify taxonomic enrichment variations among the experimental groups. Notably, significant taxonomic enrichment was observed in *A.muciniphila*, exhibited the highest LDA score of 5.324 and a *P*-value of 0.0004, as depicted in Figure 7 and Table S7.

These findings indicate that DCA exerts modulatory effects on the gut microbiota, thereby mitigating CCl_4_-induced chronic liver injury. Particularly, the substantial increase in *A.muciniphila* bacteria is suggested to play a crucial role in mediating this protective effect.

### 3.5. DCA-mediated modulation of plasma bile acid profiles via gut microbiota

To elucidate the impact of alterations in gut microbiota on metabolism via portal vein circulation, resulting in hepatic pathology, we performed plasma widely targeted metabolomic analysis on mice. Table S8 displays the metabolite information of all samples.

**Figure 8.**
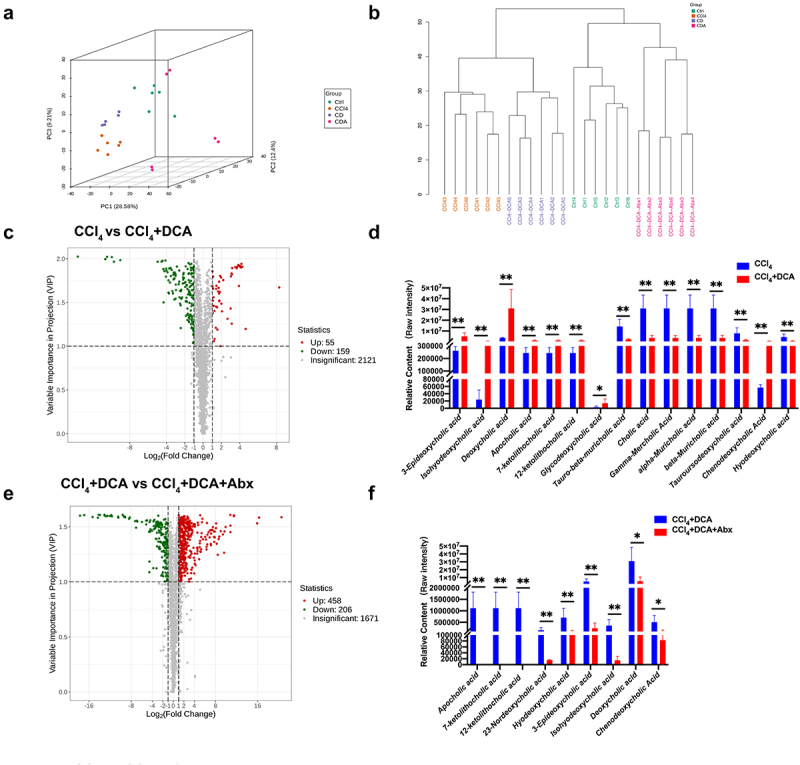
DCA regulates plasma bile acid profiles through gut microbiota (a) unsupervised PCA conducted to evaluate differences among different groups; (b) hierarchical clustering tree of samples from different groups; (c) volcano plot displaying the relative content difference of metabolites in the CCl_4_ vs. CCl_4_+DCA group; (d) comparison of plasma BAs levels between CCl_4_ and CCl_4_+DCA groups; (e) volcano plot displaying the relative content differences of metabolites in the CCl_4_+DCA and CCl_4_+DCA+Abx groups; (f) comparison of plasma BAs levels between CCl_4_+DCA and CCl_4_+DCA+Abx groups. Ctrl: Control; CD: CCl_4_+DCA; CDA: CCl_4_+DCA+Abx. *n* = 6, *P<0.05, **P<0.01, ***P<0.001, ****P<0.0001. “ns” indicates no significant difference.

Principal component analysis (PCA) and cluster analysis unveiled pronounced sample clustering within each experimental group, encompassing the Control, CCl_4_, CCl_4_+DCA, and CCl_4_+DCA+Abx groups. Importantly, the observed metabolite profiles displayed conspicuous intra-group cohesion and inter-group segregation, signifying notable disparities in the major metabolite components across the four groups (Figure 8(a,b)).

To accurately identify differentially expressed metabolites, we employed a combination of univariate and multivariate statistical analysis methods for further screening. The volcano plot demonstrated the presence of 214 differential metabolites in the plasma of the CCl_4_+DCA group when compared to the CCl_4_ group, with 55 metabolites showing upregulation and 159 metabolites showing downregulation. Among the upregulated metabolites, eight of the top 30 metabolites were BAs, while seven belonged to amino acids and their derivatives. Specifically, aside from chenodeoxycholic acid (CDCA), the upregulated BAs included 3-epideoxycholic acid, isononenebcholic acid, deoxycholic acid (DCA), apocholic acid (Apo CA) and its isomers, 12-ketolithocholic acid (12-KLCA), 7-ketolithocholic acid (7-KLCA), glycine deoxycholic acid (GDCA), and other secondary BAs. On the contrary, apart from hyodeoxycholic acid (HDCA), primary bile acids such as cholic acid (CA) and its positional isomers gamma-mercholic acid (γ-MCA), alpha-mercholic acid (α-MCA), and beta-mercholic acid (β-MCA), as well as taurine-conjugated beta-mercholic acid (*T*-β-MCA) and its positional isomer taurohyocholic acid (THCA), and tauroursodeoxycholic acid (TUDCA), all show a significant decrease. These findings indicate notable alterations in plasma BAs metabolism after DCA supplementation, particularly concerning secondary BAs. Moreover, following the administration of the antibiotic cocktail, the volcano plot demonstrated the presence of 664 differential metabolites in the plasma of the CCl_4_+DCA+Abx group when compared to the CCl_4_+DCA group, with 458 metabolites exhibiting upregulation and 206 metabolites displaying downregulation. Notably, among the BAs metabolites, there was a significant reduction observed in secondary BAs such as DCA, 3-epideoxycholic acid, apocholic acid and its isomers (12-KLCA, 7-KLCA), 23-DCA, HDCA, and IHDCA. Conversely, primary BAs did not demonstrate significant changes except for CDCA (Figure 8(c–f) and Table S9). These findings suggest that DCA influences the composition of plasma BAs through its interaction with gut microbiota, and the alterations in gut microbiota primarily impact secondary BAs rather than primary BAs.

KEGG pathway enrichment analysis of the CCl_4_ and CCl_4_+DCA groups reveals a total of 23 differentially significant metabolites (accounting for 79.31% of differentially significant metabolites annotated by KEGG.) annotated to metabolic pathways by the KEGG database. Interestingly, several differentially expressed metabolites have been additionally discovered to be enriched in the cGMP-PKG signaling pathway, the cAMP signaling pathway, and the AMPK signaling pathway, all of which are associated with the inflammatory reaction process. Differential abundance (DA) analysis reveals that, compared to the CCl_4_ group, these inflammation-related signaling pathways are significantly downregulated in the CCl_4_+DCA group. In contrast, compared to the CCl_4_+DCA group, these signaling pathways are significantly upregulated in the CCl_4_+DCA+Abx group (Supplementary Figure S3).

### 3.6. DCA modulates inflammatory signaling pathways to alleviate CCl_4_-induced chronic liver injury

**Figure 9.**
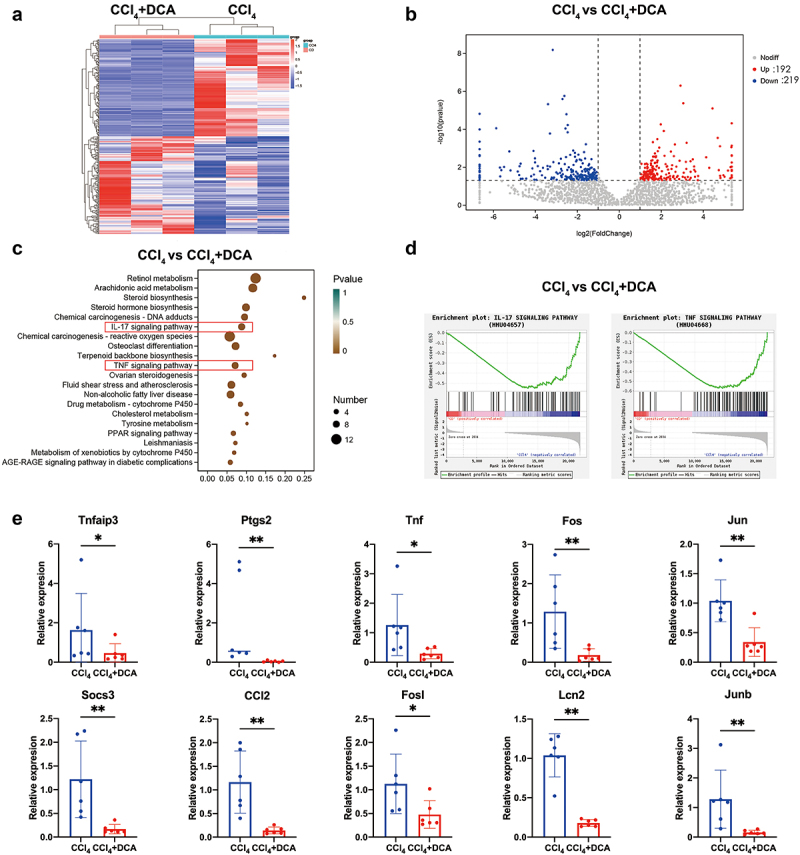
Liver transcriptome sequencing and qPCR analysis (a) cluster heatmap illustrating the gene expression in CCl_4_ and CCl_4_+DCA groups, with red indicating high-expression genes and blue indicating low-expression genes; (b) volcano plot displaying the relative content and statistical differences of genes in the CCl_4_ and CCl_4_+DCA groups; (c) gene function prediction based on the KEGG database; (d) GSEA analysis of IL-17 and TNF pathways; (e) real-time fluorescence quantitative PCR used to detect the expression of genes related to the IL-17 and TNF pathways.

To explore the relationship among changes in BAs profiles, gut microbial composition, and liver injury, we conducted transcriptome sequencing on mice liver tissue. Cluster analysis revealed significant differences in gene expression patterns between the CCl_4_+DCA and CCl_4_ groups, indicating a biological connection and exhibiting a pattern of intra-group aggregation and inter-group separation (Figure 9(a)). The volcano plot demonstrated that compared to the CCl_4_ group, 192 genes were significantly upregulated, while 219 genes were significantly downregulated in the CCl_4_+DCA group (Figure 9(b)).

Further KEGG and GSEA enrichment analysis revealed a significant downregulation of IL-17 and TNF inflammatory signaling pathways (Figure 9(c,d)). Subsequently, real-time fluorescent quantitative PCR was employed to validate the expression of genes associated with the IL-17 and TNF inflammatory pathways. The results confirmed that 10 genes (TNFAIP3, PTGS2, TNF, FOS, JUN, SOCS3, CCL2, FOSL1, LCN2, JUNB) were significantly downregulated in the CCl_4_+DCA group, coincided with the transcriptome sequencing results (Figure 9(e)). These findings suggest that DCA may inhibit the activity of IL-17 and TNF inflammatory signaling pathways, thereby attenuating CCl_4_-induced chronic liver injury through the mitigation of liver inflammation.

### 3.7. Multi-omics analysis

**Figure 10.**
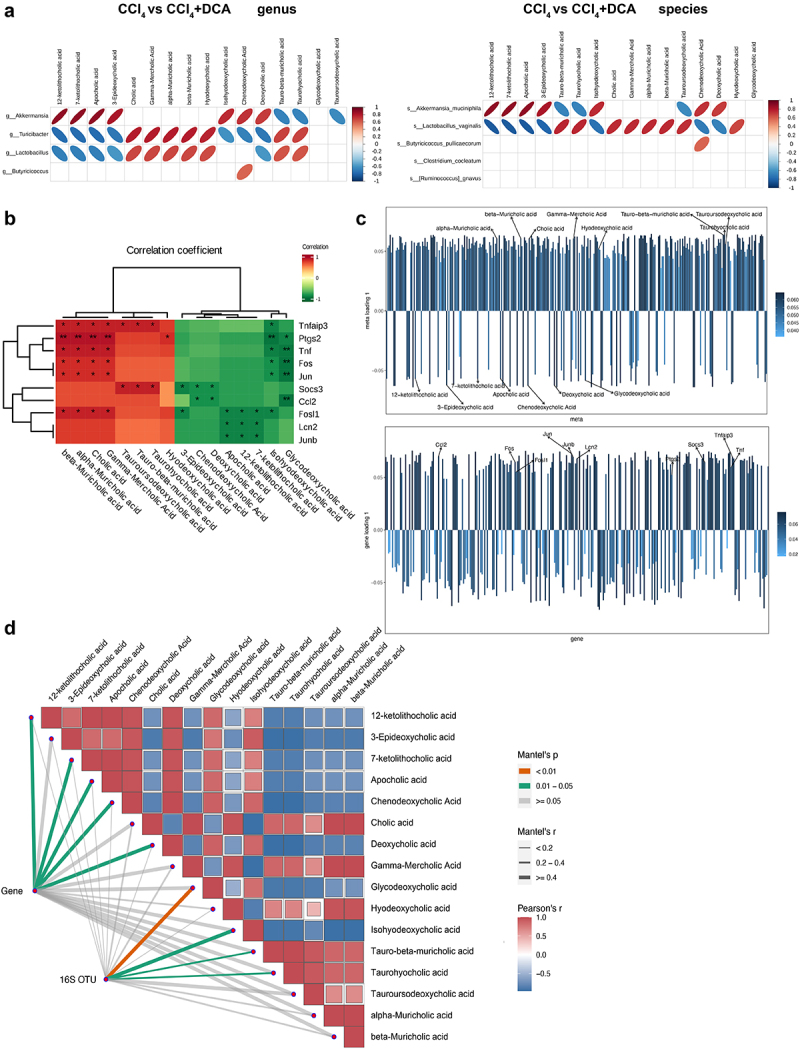
Multi-omics analysis (a) correlation heatmap of differential gut microbiota and differential BAs metabolites at the genus and species levels between CCl_4_ and CCl_4_+DCA groups; (b) correlation analysis between differential BAs metabolites and differential genes of IL-17 and TNF inflammatory signaling pathway. (c) O2PLS analysis results for metabolome and transcriptome. Bar heights reflect correlation magnitude with respective omics, darker colors signifying stronger correlations; (d) correlation analysis of differentially expressed gut microbiota and BAs metabolites between CCl_4_ and CCl_4_+DCA groups, as well as differentially expressed genes involved in IL-17 and TNF inflammatory pathways. The width corresponds to Mantel’s r statistic, representing the correlation strength, and the color represents the statistical significance of Mantel analysis.

We initially conducted a conjoint analysis to examine the relationship between differential gut microbiota and differential BAs in the CCl_4_ and CCl_4_+DCA groups (Figure 10(a)). Our analysis revealed a high positive correlation between *A.muciniphila* and secondary BAs, including 3-epideoxycholic acid (r = 0.818, *p* = 0.001), apocholic acid (Apo-CA) and its isomers 12-KLCA and 7-KLCA (r = 0.881, *p* < 0.001). Additionally, a strong positive correlation was observed between *A.muciniphila* and secondary BAs such as IHDCA (r = 0.748, *p* = 0.001) and DCA (r = 0.692, *p* = 0.013). Conversely, a strong negative correlation was found between *A.muciniphila* and primary BAs, including *T*-β-MCA and its isomers THCA (r = −0.664, *p* = 0.018) and TUDCA (r = −0.636, *p* = 0.026) (Supplementary Figure S4). These above results indicate that that DCA can regulate plasma BAs metabolism by increasing the abundance of *A.muciniphila* and promoting the production of secondary BAs.

Next, we performed correlation analysis using Spearman’s correlation test on differential BAs metabolites and differentially expressed genes in the IL-17 and TNF inflammatory pathways between CCl_4_ and CCl_4_+DCA groups. The results demonstrated a negative correlation between secondary BAs and the differential genes related to the IL-17 and TNF inflammatory pathways, while a positive correlation was observed between primary BAs and the differential genes associated with these inflammatory pathways (Figure 10(b) and Table S10). To investigate the presence of a linkage effect between transcriptomic and metabolomic profiles, O2PLS analysis was performed on the two omics datasets (Figure 10(c)). The results demonstrated a positive correlation between IHDCA, CDCA, DCA, 3-Epideoxycholic acid, GDCA, Apo CA, and its isomers 12-KLCA and 7-KLCA with transcriptomics, with length values of 0.063, 0.063, 0.062, 0.062, 0.058, and 0.057 respectively. In contrast, THCA, *T*-α-MCA, CA and its positional isomers γ-MCA, α-MCA and β-MCA, TUDCA and HDCA, exhibited a negative correlation with the transcriptome results, with length values of 0.064, 0.064, 0.061, 0.056 and 0.052. Detailed metabolite loading and length values are provided in supporting Table S11. These findings underscore the substantial impact of bile acids on transcriptomics. Similarly, in the O2PLS analysis results, JUN, CCL2, JUNB, TNFAIP3, LCN2, FOS, SOCS3, TNF, and PTGS2 were found to exhibit a negative correlation with the metabolomics results, with length values of 0.073, 0.070, 0.070, 0.069, 0.068, 0.065, 0.064, 0.060, and 0.053, respectively. Detailed gene loading and length values are provided in supporting Table S12. These findings underscore the substantial impact of IL-17 and TNF signaling pathway-associated genes on metabolomics. Subsequently, Mantel tests were conducted to integrate multi- omics data, using differential bile acids (BAs) metabolites as environmental factors. As depicted in Figure 10(d), the 16S OTUs and gene expression profiles from the two sample groups showed significant correlations with the 16 BAs metabolites. Notably: (1) DCA exhibited a strong correlation with gene expression (Mantel’s r = 0.961, p = 0.049). (2) IHDCA demonstrated a significant correlation with 16S OTUs (Mantel’s r = 0.527, p = 0.047). (3) GDCA displayed a strong correlation with gene expression (Mantel’s r = 0.602, p = 0.083). Detailed calculation results are summarized in Table S13.

These findings underscore the interconnectedness of gut microbiota, BAs metabolism, and hepatic gene expression, indicating that variations in gut microbiota play a role in modulating plasma BAs metabolism, thereby potentially influencing the expression of genes associated with IL-17 and TNF inflammatory pathways in the liver, ultimately leading to the attenuation of hepatic inflammation.

## 4. Discussion

In this study, we conducted a quantitative assessment of BAs metabolism in patients with CLD and healthy individuals. Our findings revealed a disrupted BAs metabolism profile in CLD patients, characterized by a significant reduction in secondary BAs, notably DCA, which aligns with the findings reported by Sang et al. .^8^ It has been previously demonstrated that DCA supplementation can attenuate liver injury and inflammation in murine models of liver abscess and bloodstream infections caused by *Klebsiella pneumoniae* .^6^ Nevertheless, it remains unclear whether DCA exerts its hepatoprotective effects in the context of CLD through the regulation of gut microbiota and host metabolism.

To elucidate the processes underlying the antagonistic effects of DCA on liver injury, our initial investigation focused on the impact of DCA on chronic liver injury induced by CCl_4_ in animal models. Our results demonstrated the effective mitigation of CCl_4_-induced chronic liver injury by DCA. Importantly, this protective effect was found to be dependent on the gut microbiota, as evidenced by the abolition of protection when gut microbiota was depleted using antibiotics. Subsequently, we investigated the potential mechanisms through which DCA modulates liver injury via the gut microbiota. Analysis of 16S rRNA sequencing revealed that DCA could regulate the composition of the gut microbiota, characterized by a significant increase in the Akkermansia and Butyricicoccus genera, while a notable decrease in the Turacibacter and Lactobacillus genera was observed. Notably, the most prominent effect was observed in *A. muciniphila*.

*A.muciniphila*, a probiotic gut microbiota, has been recognized for its potential to improve obesity, metabolic disorders, and modulate host immunity.^21^ Previous studies have reported its ability to induce beneficial changes in gut microbiota composition by altering diversity and richness, resulting in improvements in immune-mediated liver diseases.^22^ Research has shown that various bile acid (BA) salts have distinct effects on the growth of *A. muciniphila*. While most BA salts inhibit its growth, sodium deoxycholate can stimulate it, a finding consistent with our research results. This stimulatory effect may be due to *A. muciniphila*‘s ability to protect its squalene-related membranes from BA-induced damage.^23^

Interestingly, we observed that DCA not only significantly enhanced the growth of *A.muciniphila* but also induced alterations in host BAs metabolism, specifically increasing secondary BAs while decreasing primary BAs. When we depleted gut microbiota, *A. muciniphila* abundance decreased, along with a reduction in secondary BAs, while primary BAs remained relatively unchanged. Correlation analysis revealed a strong positive relationship between *A. muciniphila* and several secondary BAs, such as 3-epideoxycholic acid, apocholic acid (Apo-CA), its isomers 12-KLCA and 7-KLCA, isohyodeoxycholic acid (IHDCA), and deoxycholic acid (DCA). These findings suggest that *A. muciniphila*, influenced by DCA treatment, plays a role in altering secondary BA metabolism. Consequently, *A. muciniphila* may induce alterations in the bile acid pool by modulating bile acid metabolism, ultimately mitigating liver inflammation injury. Similarly, several previous studies have indicated that *A. muciniphila* plays a significant role in improving liver health by regulating bile acid metabolism.^24,25^ There may be two potential reasons supporting these observations. Firstly, *A. muciniphila* can enhance gut lipid oxidation, reduce oxidative stress, and inhibit cell apoptosis associated with lipid accumulation, stabilizing BAs metabolism. Furthermore, *A. muciniphila* can thicken the mucus layer in the gut, reducing permeability and reversing gut barrier damage, which in turn preserves gut barrier function.^26^ However, the observed association between *A. muciniphila* and BAs may not firmly establish the direct involvement of *A. muciniphila* in improving liver inflammation through BAs regulation. To confirm this, additional fecal transplant experiments are necessary to directly demonstrate the impact of A. muciniphila on bile acids and liver inflammation in the future.

Interestingly, as previously mentioned, we observed a significant decrease in the abundance of Turicibacter and *Lactobacillus vaginalis* in mice treated with DCA, suggesting their potential involvement in BAs metabolism regulation. However, it is important to note that the subsequent gut microbial depletion experiments could not confirm the role of these two bacteria in plasma BAs metabolism, as their abundance decreased significantly following DCA and continued to decrease after Abx treatment. Additionally, the magnitude of change and abundance of these two bacteria were considerably lower compared to *A.muciniphila*, suggesting that the modulation of BAs profiles in plasma is primarily mediated by *A.muciniphila*.

The analysis of the gut microbiome and metabolomics suggests that the administration of DCA increases secondary bile acids in plasma through the modulation of gut microbiota. Next, we further explored how elevated secondary bile acids affect the processes of liver injury. Our liver transcriptome sequencing revealed that DCA downregulates the IL-17 and TNF inflammatory pathways. Conjoint analysis demonstrated a connection between BAs metabolism and these inflammatory pathways, suggesting that secondary bile acids may mitigate inflammation by inhibiting gene expression in these pathways. Furthermore, plasma metabolomics KEGG enrichment analysis indicated a significant downregulation of inflammatory pathways, such as cGMP PKG, cAMP, and AMPK, following DCA treatment. Previous studies have documented that bile acids, specifically DCA and LCA, can suppress inflammation by activating the macrophage bile acid receptor TGR5 and utilizing cAMP to inhibit the NLRP3 inflammasome, thereby exerting anti-inflammatory effects.^27^ Importantly, it is noteworthy that the NLRP3 inflammasome contributes to liver damage through the IL-17 and TNF pathways.^28^ These findings align with our own research, further reinforcing the notion that modulating bile acid metabolism can effectively mitigate CCl_4_-induced chronic liver damage.

Building on the aforementioned findings, we propose that DCA exerts its protective effects against CCl_4_-induced chronic liver injury through the following processes: DCA significantly increases the abundance of *A.muciniphila* in the gut microbiota, thereby modulating the BAs profile and antagonizing chronic liver injury induced by CCl_4_. The mechanism may involve the inhibition of hepatic IL-17 and TNF signaling pathway expressions.

BAs play a crucial role in the enterohepatic circulation, and their interaction with gut microbiota is bidirectional. On one hand, elevated levels of BAs can disrupt gut barrier function, promote bacterial translocation, and activate hepatic inflammation and fibrosis.^29^ On the other hand, gut microbiota actively participate in BAs metabolism,^30^ influencing their metabolism and biological activities through fermentation, transformation, oxidation, reduction, and other processes. Certain gut microbiota can hydrolyze or deoxygenate BAs, leading to the production of secondary BAs. These secondary BAs exhibit diverse metabolic pathways and biological activities, potentially impacting liver health. Moreover, the gut microbiota can influence the enterohepatic circulation of BAs by regulating gut barrier function, immune modulation, antioxidant and anti-inflammatory effects, and other mechanisms. Therefore, investigating the interplay between the gut microbiota and BAs is crucial for understanding the pathogenesis of liver diseases and developing relevant therapeutic strategies. In our study, we demonstrated that DCA, a secondary BAs, effectively antagonizes CCl_4_-induced chronic liver injury. This effect is associated with a significant increase in *A.muciniphila*, which modulates BAs metabolism and promotes the conversion of primary BAs to secondary BAs. Future research should delve into the mechanisms by which DCA regulates BAs metabolism through gut microbiota. Building upon this knowledge, targeting gut microbiota could offer new avenues for the treatment of chronic liver diseases.

Nevertheless, we acknowledge several limitations of this study. Firstly, the construction of a pseudo-sterile mouse model using an antibiotic cocktail may not achieve true sterility compared to truly sterile mice. Residual resistant microorganisms may still be present, and future studies can explore this aspect using truly sterile mice. Secondly, our study demonstrates a significant correlation among bile acid metabolism, chronic liver injury, and the gut microbiota. However, establishing causality requires further investigation. Future research should delve into mechanistic pathways to elucidate how the gut microbiota mediates DCA modulation of BAs metabolism.

## Supplementary Material

Supplemental Material

## Data Availability

The datasets that support the findings of the study are available from the corresponding author upon reasonable request. The 16S rRNA gene sequencing data from this study are available in the NCBI Sequence Read Archive (SRA) repository under the accession number PRJNA1031407.
