# Supplementary material for: Multi-omics reveals deoxycholic acid modulates bile acid metabolism via the gut microbiota to antagonize carbon tetrachloride-induced chronic liver injury
Source: Gut Microbes. 2024 Feb 28;16(1):2323236. doi: 10.1080/19490976.2024.2323236 (PMC10903553; doi:10.1080/19490976.2024.2323236)
Supplement: Supplemental Material [file KGMI_A_2323236_SM2558.zip › Supplementary document.docx]

**﻿Supplementary Information for**

**Multi-omics reveals deoxycholic acid modulates bile acid metabolism via the gut microbiota to antagonize carbon tetrachloride-induced chronic liver injury**

Li Zhang ^1, 2, 3, 4^, Zhiyi Zheng^1, 2, 3, 4^, Huanhuan Huang^5^, Ya Fu^1, 2, 3, 4^, Tianbin Chen^1, 2, 3, 4^, Can Liu^1, 2, 3, 4^, Qiang Yi^1, 2, 3, 4^, Caorui Lin^1, 2, 3, 4^, Yongjun Zeng^6^, Qishui Ou^1, 2, 3, 4, *^ Yongbin Zeng ^1, 2, 3, 4*^

1 Department of Laboratory Medicine, Gene Diagnosis Research Center, the First Affiliated Hospital, Fujian Medical University, Fuzhou 350005, China.

2 Department of Laboratory Medicine, National Regional Medical Center, Binhai Campus of the First Affiliated Hospital, Fujian Medical University, Fuzhou 350212, China.

3 Fujian Key Laboratory of Laboratory Medicine, the First Affiliated Hospital, Fujian Medical University, Fuzhou 350005, China.

4 Fujian Clinical Research Center for Laboratory Medicine of Immunology, the First Affiliated Hospital, Fujian Medical University, Fuzhou 350005, China

5 Department of Pediatrics, the First Affiliated Hospital, Fujian Medical University, Fuzhou 350005, China

6 Department of Cardiology, the First Affiliated Hospital, Fujian Medical University, Fuzhou 350005, China

Word count: 8018

* Corresponding author.

E-mail addresses: ouqishui@fjmu.edu.cn (Qishui Ou), zengyongbin@fjmu.edu.cn (Yongbin Zeng)

Li Zhang: zl13159467955@163.com; Zhiyi Zheng: 757078284@qq.com; Huanhuan Huang: [huanghuanhuan@fjmu.edu.cn](mailto:huanghuanhuan@fjmu.edu.cn); Ya Fu: 15280429582@163.com; Tianbin Chen: nihaochtb@126.com; Can Liu: [liucan1012@163.com](mailto:liucan1012@163.com); Qiang Yi: yiqiang1499@163.com; Caorui Lin: m13652171863@163.com; Yongjun Zeng: zengyongjun@fjmu.edu.cn; Qishui Ou: ouqishui@fjmu.edu.cn; Yongbin Zeng: zengyongbin@fjmu.edu.cn.

﻿**﻿Table of contents:**

1. Supplementary Methods

2. Supplementary Figures

**1.1 The HPLC conditions and ESI-MS/MS conditions for detection of bile acids**

**1.1.1 HPLC Conditions**

The sample extracts were analyzed using an LC-ESI-MS/MS system (UHPLC, ExionLC™ AD, https://sciex.com.cn/; MS, Applied Biosystems 6500 Triple Quadrupole, https://sciex.com.cn/). The analytical conditions were as follows, HPLC: column, Waters ACQUITY UPLC HSS T3 C18 (100 mm×2.1 mm i.d., 1.8 µm); solvent system, water with 0.01% acetic acid and 5 mmol/L ammonium acetate (A), acetonitrile with 0.01% acetic acid (B); The gradient was optimized at 5% to 40% B in 0.5 min, then increased to 50% B in 4 min, then increased to 75% B in 3 min, and then 75% to 95% in 2.5min, washed with 95%B for 2 min ,finally ramped back to 5% B (12-14 min); flow rate, 0.35 mL/min; temperature, 40°C; injection volume: 3 μL. The effluent was alternatively connected to an ESI-triple quadrupole-linear ion trap (QTRAP)-MS.

**1.1.2 ESI-MS/MS Conditions**

Linear ion trap (LIT) and triple quadrupole (QQQ) scans were acquired on a triple quadrupole-linear ion trap mass spectrometer (QTRAP), QTRAP® 6500+ LC-MS/MS System, equipped with an ESI Turbo Ion-Spray interface, operating in negative ion mode, and controlled by Analyst software (version 1.6.3). The ESI source operation parameters were as follows: ion source, ESI-; source temperature 550 ℃; ion spray voltage (IS) -4500 V; curtain gas (CUR) was set at 35 psi, respectively. Bile acids were analyzed using scheduled multiple reaction monitoring (MRM). Data acquisitions were performed using Analyst software (version 1.6.3). MultiQuant software (version 3.0.3) was used to quantify all metabolites. Mass spectrometer parameters including the declustering potentials (DP) and collision energies (CE) for individual MRM transitions were done with further DP and CE optimization. A specific set of MRM transitions were monitored for each period according to the metabolites eluted within this period.

**1.2 The HPLC conditions, QTOF-MS/MS, and ESI-Q TRAP-MS/MS conditions for detection of metabolite**

**1.2.1 T3 UPLC Conditions**

The sample extracts were analyzed using an LC-ESI-MS/MS system (UPLC, ExionLC AD, https://sciex.com.cn/; MS, QTRAP® System, https://sciex.com/). The analytical conditions were as follows, UPLC: column, Waters ACQUITY UPLC HSS T3 C18 (1.8μm, 2.1 mm*100 mm); column temperature, 40°C; flow rate, 0.4 mL/min; injection volume, 2μL or 5μL; solvent system, water (0.1% formic acid): acetonitrile (0.1% formic acid); gradient program, 95:5 V/V at 0 min, 10:90 V/V at 10.0 min, 10:90 V/V at 11.0 min, 95:5 V/V at 11.1 min, 95:5 V/V at 14.0 min.

**1.2.2 QTOF-MS/MS**

The Triple TOF mass spectrometer was used for its ability to acquire MS/MS spectra on an information-dependent basis (IDA) during an LC/MS experiment. In this mode, the acquisition software (TripleTOF 6600, AB SCIEX) continuously evaluates the full scan survey MS data as it collects and triggers the acquisition of MS/MS spectra depending on preselected criteria. In each cycle, 12 precursor ions whose intensity greater than 100 were chosen for fragmentation at collision energy (CE) of 30 V (12 MS/MS events with product ion accumulation time of 50 msec each). ESI source conditions were set as following: Ion source gas 1 as 50 Psi, Ion source gas 2 as 50 Psi, Curtain gas as 25 Psi, source temperature 500°C, Ion Spray Voltage Floating (ISVF) 5500 V or -4500 V in positive or negative modes, respectively.

**1.2.3 ESI-Q TRAP-MS/MS**

LIT and triple quadrupole (QQQ) scans were acquired using a triple quadrupole-linear ion trap mass spectrometer (QTRAP), specifically the QTRAP® LC-MS/MS System. This system is equipped with an ESI Turbo Ion-Spray interface and operates in both positive and negative ion modes. The entire system is controlled by Analyst software (version 1.6.3). The ESI source operated under the following parameters: source temperature at 500°C; ion spray voltage (IS) set at 5500 V (positive) and -4500 V (negative); ion source gas I (GSI), gas II (GSII), and curtain gas (CUR) were maintained at 50, 50, and 25.0 psi, respectively. The collision gas (CAD) was set to high. Instrument tuning and mass calibration were carried out using 10 and 100 μmol/L polypropylene glycol solutions in QQQ and LIT modes, respectively. Specific sets of MRM transitions were monitored for each period based on the elution profiles of metabolites during those periods.

**2. Supplementary Figures**

**
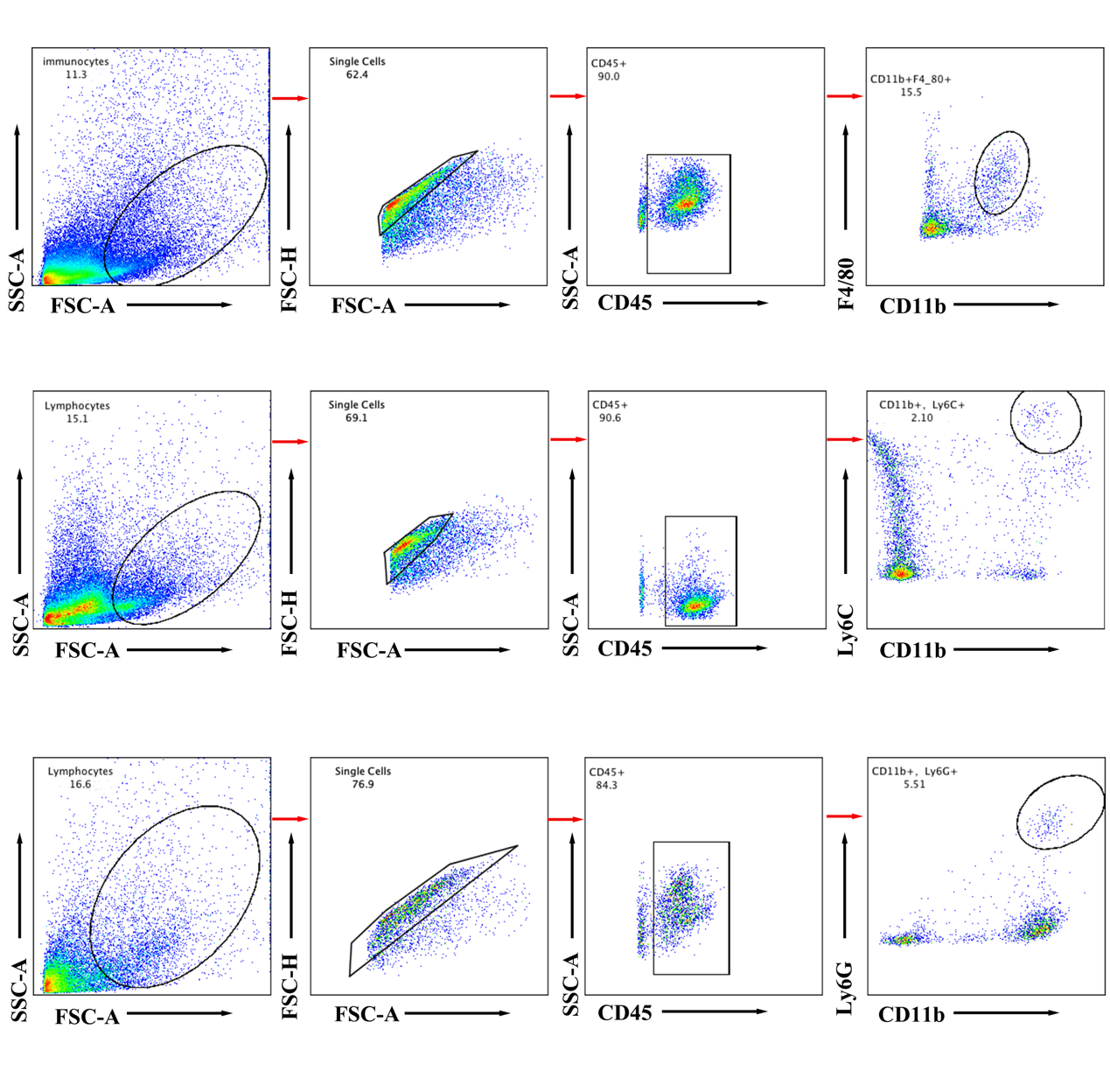
**

**Supplementary Figure 1.** The gating strategy used for the flow cytometry analysis.


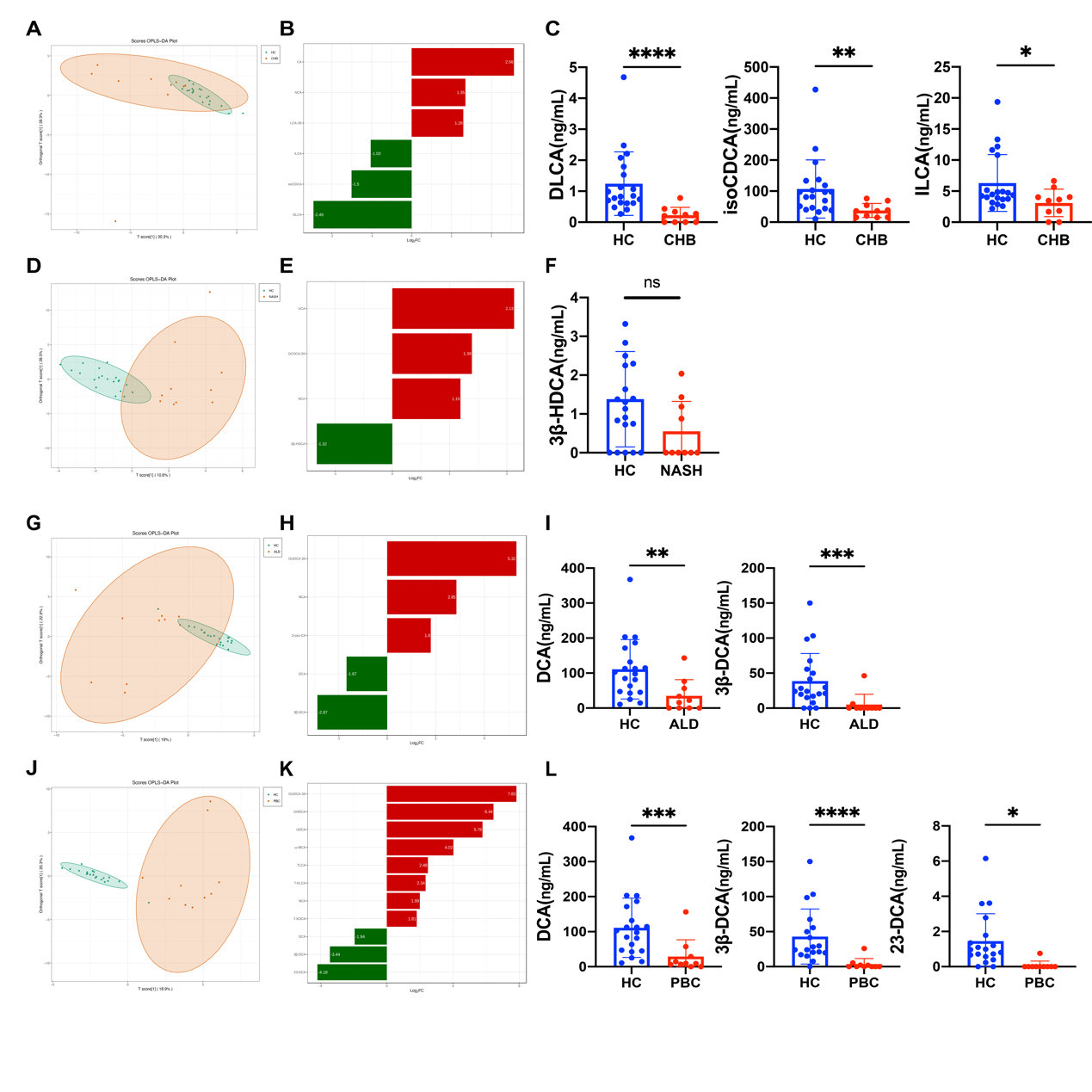


**Supplementary Figure 2.** Analysis of BAs relevant variables in healthy control and different etiologies of liver injury. (A) OPLS-DA score plot between the CHB and HC groups; (B) Bar chart of metabolites difference between CHB and HC groups; (C) Histograms of DLCA, isoCDCA and ILCA levels between CHB and HC groups; (D) OPLS-DA score plot between NASH and HC groups; (E) Bar chart of metabolites difference between NASH and HC groups; (F) Histogram of 3β-HDCA level between NASH and HC groups; (G) OPLS-DA score plot between ALD and HC groups; (H) Bar chart of metabolites difference between ALD and HC groups; (I) Histograms of 3β-DCA and DCA levels between ALD and HC groups; (J) OPLS-DA score plot between PBC and HC groups; (K) Bar chart of metabolites difference between PBC and HC groups; (L) Histograms of DCA, 3β-DCA and 23-DCA levels between PBC and HC groups. HC: Healthy control (n=20); NASH: Nonalcoholic steatohepatitis (n=10); PBC: Primary biliary cholangitis (n=10); ALD: Alcoholic liver disease (n=10); CHB: Chronic hepatitis B; CLD: Chronic liver disease (n=40), **P*＜0.05, ***P*＜0.01, ****P*＜0.001, *****P*＜0.0001


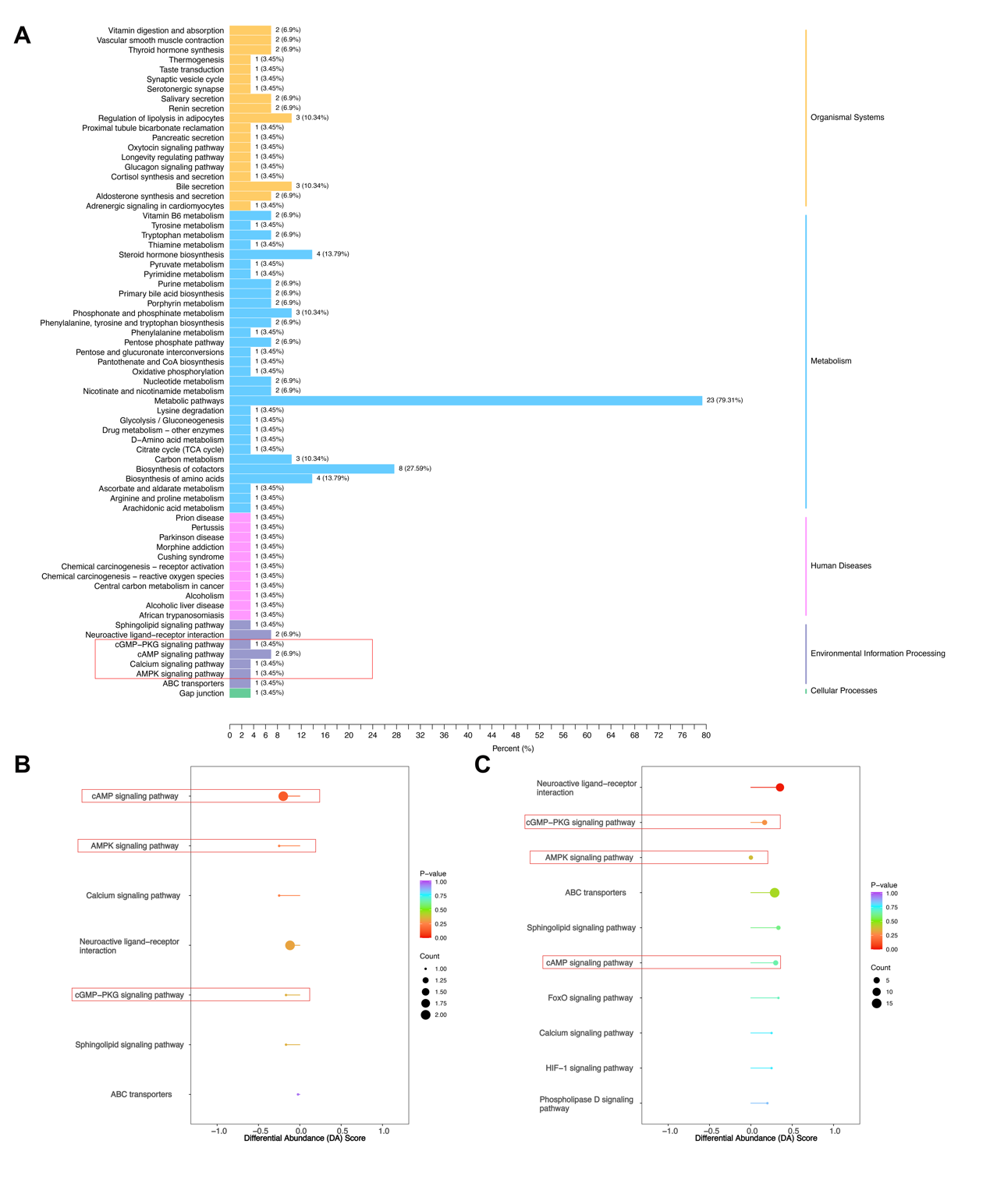


**Supplementary Figure 3.** Classification map of differential metabolite KEGG pathway. (A) The diagram of classification categorizing significantly different metabolites based on the pathway types in KEGG. The vertical axis represents the name of the KEGG metabolic pathway, and the horizontal axis represents the number of differential metabolites annotated under this pathway and their proportion to the total number of differential metabolites annotated; (B) DA score for overall changes in KEGG metabolic pathway analysis in CCl_4_ and CCl_4_+DCA groups; (C) DA scores of KEGG enriched inflammatory related pathways in the CCl_4_+DCA and CCl_4_+DCA+Abx groups.


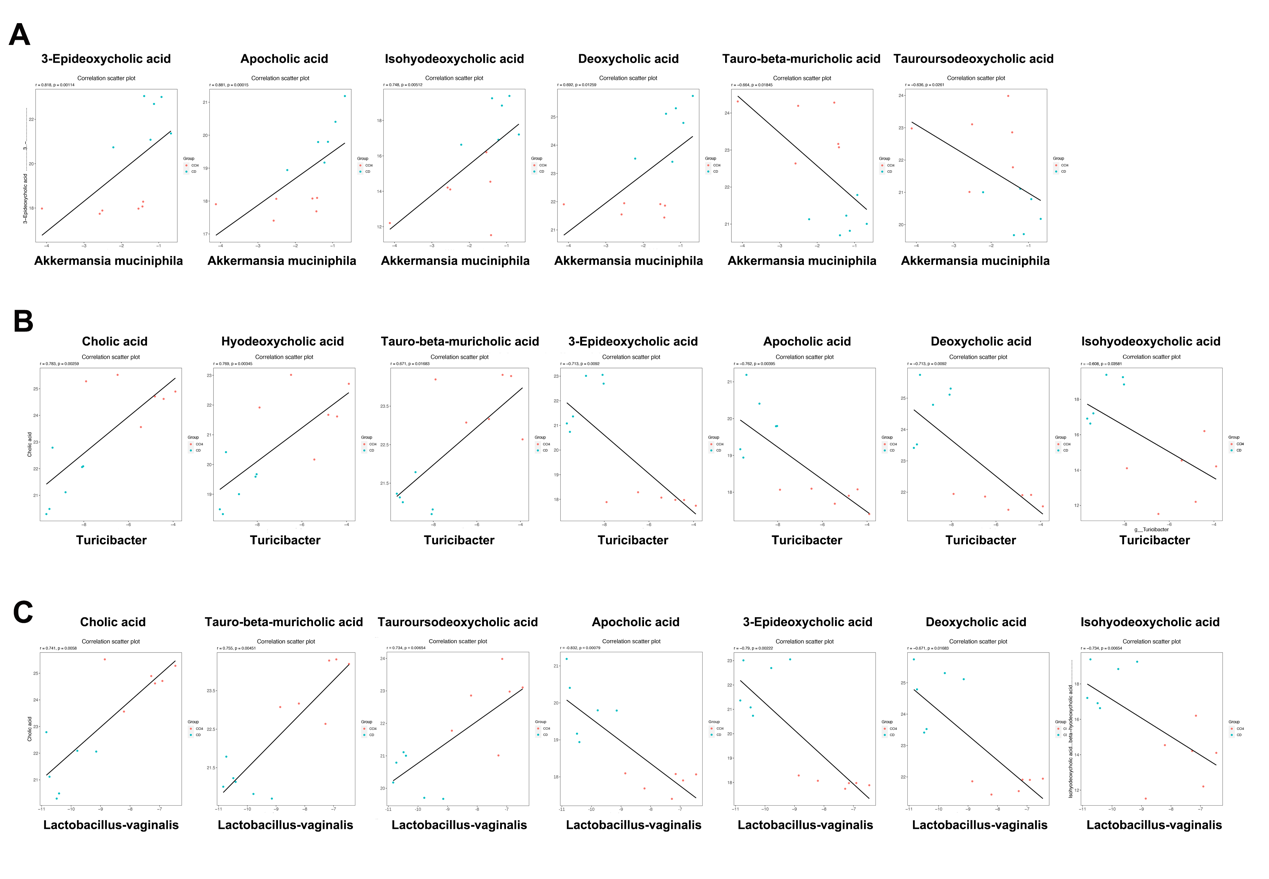


**Supplementary Figure 4.** Scatter plot of representative correlation between differential gut microbiota and differential BAs metabolites in the CCl_4_ and CCl_4_+DCA groups.
